# Usefulness of an accelerometer-based navigation system in bilateral one-stage total knee arthroplasty

**DOI:** 10.1186/s12891-021-04027-9

**Published:** 2021-02-10

**Authors:** Artit Laoruengthana, Piti Rattanaprichavej, Thanawat Tantimethanon, Watcharapong Eiamjumras, Passakorn Teekaweerakit, Krit Pongpirul

**Affiliations:** 1 Department of Orthopaedics, Faculty of Medicine, Naresuan University, 99 Moo 9 Thapho, Phitsanulok, 65000 Thailand; 2 Department of Preventive and Social Medicine, Faculty of Medicine, Chulalongkorn University, Bangkok, Thailand; 3 Department of International Health, Johns Hopkins Bloomberg School of Public Health, Baltimore, MD USA

**Keywords:** Accelerometer-based navigation, Bilateral total knee arthroplasty, Blood loss, Postoperative pain, Mechanical axis

## Abstract

**Background:**

Bilateral one-stage total knee arthroplasty (BTKA) have increased because it provides a number of advantages. Recently, Accelerometer-based navigation (ABN) system which guide the cutting plane without intramedullary disturbance might result in less endothelial and microvascular damage. Therefore, we hypothesized that the ABN may reduce blood loss, reduce postoperative pain, and better restore BTKA alignment compared to conventional instruments.

**Methods:**

We retrospectively compared 44 consecutive patients receiving ABN assisted BTKA (iBTKA) to 57 patients with conventional instruments (cBTKA). Identical pre- and post-operative care was utilized to all patients. The outcome measures assessed were hemoglobin (Hb), calculated blood loss (CBL), blood transfusion, VAS score for pain, morphine consumption, knee flexion angle, and length of stay (LOS). Radiographic assessment included mechanical axis (MA) and component positioning at 3–6 months of follow up.

**Results:**

Both iBTKA and cTKA groups had equivalent demographic data. Postoperative Hb of the cBTKA group was significantly lower than those in the iBTKA group at 24 h (*p* = 0.02), but there was no significant difference in drain volume, CBL, and blood transfusion rate. For radiographic measures, the iBTKA group had more accurate MA and component orientation, and had a lower number of outliers than those in the cBTKA group (*p* ≤ 0.01), except for the sagittal femoral component angle.

**Conclusion:**

The ABN assisted BTKA could not reduce blood loss or postoperative pain more than cBTKA, nor improve functional recovery. However, the ABN significantly improved the accuracy of MA and prostheses positioning**.**

**Trial registration:**

The protocol of this study was registered in the Thai Clinical Trials Registry database No. TCTR20180731001# on 25 July 2018.

## Background

Currently, bilateral one-stage total knee arthroplasty (BTKA) procedures have increased as an alternative option for patients with bilateral involvement [1]. However, there are potential disadvantages concerning BTKA [2], one of which is that it is associated with greater blood loss than the unilateral total knee arthroplasty (UTKA). This significant amount of blood loss may increase intra-articular pressure and swelling of soft tissue which can increase postoperative pain and require higher muscle strength to perform postoperative rehabilitation [3, 4]. Recent published studies showed approximately 1 l of calculated blood loss (CBL) and approximately 30% of allogeneic blood transfusion rate after BTKA, even if tranexamic acid (TXA) was administered [5]. Currently, meta-analysis evaluation of the effects of CAS on post-TKA bleeding demonstrated that CAS could be effective in reducing CBL by 185.4 ml as compared to conventional TKA [6]. Thus, emerging technologies that minimize medullary cavity disturbance may help mitigating these potential drawbacks of BTKA [7].

Recently, Accelerometer-Based Navigation (ABN) systems; handheld navigation systems, combine the alignment accuracy of large computer console systems, and ease of use with 2 wireless pods attached to the conventional instruments. The ABN detects the rotatory center location through a stop-and-go movement, and, following a series of short calibration steps, these gyroscopic pods display alignment information and guide the cutting plane within the operative field [8]. Goh et al. [9] demonstrated that ABN has a comparable number of outliers for the mechanical axis (MA) and coronal prostheses orientation, as compared to non CT-based CAS. In addition to improving limb alignment, another possible benefit using ABN in BTKA is that it may cause less endothelial and microvascular damage by avoiding intramedullary disturbance, and may result in less post-operative hemarthrosis and blood loss, less inflammation, lower postoperative pain intensity, and reduced potential risks related to marrow embolism [7, 10, 11]. Kawaguchi et al. [12] retrospectively reviewed 64 patients who underwent conventional unilateral TKA (32 patients) and ABN assisted TKA (32 patients). They found that the patients in the ABN group had significantly less estimated blood loss when compared to patients using conventional instruments (422.5 ± 187.5 vs 563.6 ± 261.9; *p* < 0.01).

Hence, we hypothesized that using ABN would improve the accuracy of postoperative MA and component positioning, and may solve some concerning issues related to conventional BTKA. Because of limited studies for ABN in BTKA, we conducted the present study to determine whether ABN can 1) reduce blood loss, 2) reduce postoperative pain, 3) better restore mechanical and prosthesis alignment, and 4) reduce complications after BTKA when compared to conventional system.

## Methods

This investigation was a retrospective, cohort study of patients who had undergone BTKA with either conventional instruments (cBTKA) or accelerometer-based navigation (iBTKA) by a single, senior arthroplasty surgeon (AL) who expertise in both conventional and iASSIST™ system. All patients with a diagnosis of primary osteoarthritis of both knees and who were undergoing bilateral TKA in the same anesthesia were enrolled. Exclusion criteria included patients with prior knee surgery history, prior knee infection, and hip pathology that severely limited range of motion. Forty-four consecutive patients who received iBTKA during 2018–2019 and a cohort of 57 patients that underwent cBTKA during 2016–2018 were compared. The study was approved by the Institutional Review Board, and informed consent was elicited from every patient.

Regional anesthesia comprising Bupivacaine (0.5% Marcaine, AstraZeneca, Sweden), prophylactic antibiotic injection, and tourniquet control at 250 mmHg, were applied to all patients. All the surgical procedures were performed by a single surgeon via standard medial parapatellar arthrotomy. For the cBTKA group, the proximal tibial bone was first cut using an extramedullary alignment guide which was targeting perpendicularly to the tibial coronal axis. The distal femoral cut was then done with intramedullary reference guides aiming for 5° of the valgus in coronal plane, and aiming perpendicularly to the anatomical axis in sagittal plane. Femoral entry point was altered in coronal plane according to the anatomical axis as preoperatively template, while the entry point in sagittal plane was set at 1 cm anterior to the top of intercondylar notch which allow the intramedullary guide aligned to sagittal femoral axis [13]. The posterior condylar axis was used as a reference for 3° external rotation of the femoral component. Likewise, the iBTKA group received identical anesthetic and surgical procedures except that the ABN system (iASSIST™, Zimmer inc., Warsaw, IN, USA) was applied to guide proximal tibial and distal femoral bone cuts. For the iBTKA group, the femoral spike was impacted at the similar entry point of the distal femur, and the reference pod was attached to the spike. The leg was then moved around the hip center to orientate the MA of the femur, and the distal femoral cut was made perpendicularly to the mechanical alignment in coronal plane and 1^o^ of flexion from the MA in sagittal plane. For femoral rotation, the anterior reference guide was used to avoid anterior femoral notching, and posterior condyles were the reference axis to set 3° external rotation of the femoral component. For the tibia, the reference pod was attached to the extramedullary guide, and the leg was then moved into the abduction and adduction direction over the ankle center. The tibial resection was also performed perpendicularly to the MA in coronal plane and was set for 5° of the posterior tibial slope. A validation tool was used to confirm both femoral and tibial cut orientation, and re-resection would be done if the cuts deviated > ± 1^o^ of the aiming target either in the coronal or sagittal plane. The patella was not resurfaced in both groups. The intramedullary opening of the femur was occluded with a bone plug in all knees of cBTKA group. A periarticular injection (PAI) mixture consisting of 100 mg of Bupivacaine (0.5% Marcaine; AstraZeneca, Sweden), 30 mg of ketorolac tromethamine (ketorolac tromethamine 1 ml; SiuGuan, Taiwan), and sterile normal saline solution, was divided to be injected into bilateral knees. All patients in both groups received cemented, fixed-bearing, posterior stabilized prostheses (NexGen LPS, Zimmer Biomet, Warsaw, IN, USA). Before the arthrotomy closure, a suction drain was applied into the knee joint, and tranexamic acid (15 mg/kg) was poured into the joint. The drain was clamped for 3 h, and a compressive dressing was applied following the arthrotomy closure. Afterward, the drain and dressing was removed at 24 h after the surgery.

Identical postoperative care was provided to all patients. For the first 48 h, an intravenous patient-controlled analgesia (PCA) morphine (100 ml solution containing 50 mg of morphine sulphate) was injected as an on-demand bolus of 1 ml with a 5 min lockout period, 30 mg of ketorolac was given intravenously every 8 h, and 500 mg of oral acetaminophen was administered three times a day. Thereafter, all the catheters were discarded, and then 250 mg of naproxen was given orally twice a day, as well as 500 mg of acetaminophen was given orally three times a day. Two milligrams of morphine were additionally used for breakthrough pain every 4 h throughout hospitalization. Rehabilitation and a continuous passive motion (CPM) device were utilized for all patients to promote early ambulation with gait aids. All patients were administered with low molecular weight heparin for the first 48 h, and combined with oral warfarin for 10 days.

The standard postoperative long-leg anteroposterior (AP) radiograph (digitalized) in weight bearing with fully extend knee and lateral weight-bearing knee film were obtained preoperative, and at 3–6 months of follow up. Patients were placed on a platform to visualize the ankle joint, while toes pointed forward and lateral malleoli was 20 cm apart. As a rotation control, the patella was placed perpendicular to the x-ray beam. Only adequate radiograph without evidence of malrotation was analyzed. Four radiographic parameters were measured [14, 15]: (A) lower extremity MA; (B) coronal femoral component angle (CFA); (C) coronal tibial component angle (CTA); (D) sagittal femoral component angle (SFA); (E) sagittal tibial component angle (STA) or tibial slope. The coronal MA was determined by the line connecting the center of the femoral head and center of the ankle [14]. The MA is subdivided into the femoral MA, which is the line running from hip center through the center of intercondylar notch of the distal femur (or femoral prosthesis), and the tibial MA, which extends from the center of tibial plateau (or tibial tray) to the center of the talus [14, 15]. A negative and positive value indicated a valgus and varus deformity, respectively. Postoperative CFA was defined as the angle formed by a line connecting the distal part of the medial and lateral condyles of the femoral component and the MA of femur. The angle that was formed by the MA of the tibia and a line parallel to the surface of the tibial tray, was determined as CTA. The angle >90^o^ was defined as a valgus position, whereas the angle <90^o^ represented a varus position. For the lateral weight-bearing knee film, the distal femoral and proximal tibial anatomical axes were set as the line connecting the midpoints of the outer cortex 5 cm and 15 cm proximal and distal to the joint line, respectively. The SFA and STA was defined as the angle formed by the line parallel to the distal femoral implant and the anatomical axis of femur, and a line parallel to the tibial baseplate and anatomical axis of tibia, respectively [15, 16]. The angle >90^o^ indicated an extension position, while the angle <90^o^ was referred to a flexion position.

Outlier was defined as the alignment that deviating > ± 3° from the satisfactory parameters which were 0° of lower extremity MA, 90° of CFA, 90° of CTA, 90° of SFA, and 85^o^ of STA (or 5^o^ of posterior tibial slope). The radiographs were evaluated by three assessors who were blind to the study group. All these parameters were compared between the two groups. Other outcomes including operative time, drain volume, hemoglobin (Hb), calculated blood loss (CBL), allogeneic blood transfusion, intensity of pain determined by the 10-cm visual analog scale (VAS), morphine consumption, maximal angle of knee flexion that the patient could tolerate as measure by the CPM device, and length of stay (LOS) that were prospectively collected at our institution, were also compared between groups.

The patient’s total blood volume (TBV) was calculated using the equation of Nadler et al. [17]. The difference between preoperative and lowest postoperative Hb was applied with the hemoglobin balance method to determine CBL [18], which was accounted as the sum of external blood loss and hidden blood loss (extravasation into the soft tissues and loss due to hemolysis) [19].

$$ \mathrm{Male}:\mathrm{TBV}\ \left(\mathrm{ml}\right)=\left(0.0003669\ \mathrm{x}\ {\mathrm{height}}^3\left[\mathrm{cm}\right]\right)+\left(32.19\ \mathrm{x}\ \mathrm{body}\ \mathrm{weight}\ \left[\mathrm{kg}\right]\right)+604 $$

$$ \mathrm{Female}:\mathrm{TBV}\ \left(\mathrm{ml}\right)=\left(0.0003561\ \mathrm{x}\ {\mathrm{height}}^3\left[\mathrm{cm}\right]\right)+\left(33.08\ \mathrm{x}\ \mathrm{body}\ \mathrm{weight}\ \left[\mathrm{kg}\right]\right)+183 $$

$$ {\displaystyle \begin{array}{l}\mathrm{Calculated}\ \mathrm{blood}\ \mathrm{loss}\ \left(\mathrm{ml}\right)=\mathrm{TBV}\ \left[\mathrm{ml}\right]\ \mathrm{x}\ \left({\mathrm{Hb}}_{\mathrm{i}}-{\mathrm{Hb}}_{\mathrm{e}}\right)/{\mathrm{Hb}}_{\mathrm{i}}+\mathrm{sum}\ \mathrm{of}\ \mathrm{blood}\ \mathrm{products}\\ {}\mathrm{transfused}\ \left[\mathrm{ml}\right],\end{array}} $$

Hb_i_ [g/dl] was defined as the preoperative Hb, and Hb_e_ [g/dl] was the lowest postoperative Hb.

A serum Hb level below 9.0 g/dl is rountinely indicated for a blood transfusion in BTKA at our institution. All of the clinical assessors were blinded to the treatment protocol.

### Statistical analysis

All measured characteristics and outcomes were summarized with descriptive statistics. Data were checked with Kolmogorov – Smirnov tests for normality. Continuous data are presented as mean and standard deviation, and were compared between groups using the Student’s *t* test (normal distribution data) or the Mann-Whitney *U* test (non-normally distributed data). Categorical data are presented as counts and percentages, and were compared by Fisher’s exact test. Repeated measures ANOVA was also used to compare the time-dependent data including change of postoperative Hb, VAS, and knee flexion angle between groups. The post hoc comparisons of all pairwise of points in time were applied to account for multiple testing with Bonferroni adjustments. The sample size of iTKA and cTKA groups had 91.3% power to detect a difference of 200 mL in CBL, which could significantly impact on blood transfusion rate, with standard deviation (SD) of 300 mL [20]; 84.8% power to ascertain the minimal clinically important difference (MCID) of 1.5 for VAS with SD of 2.0 [21]; and 91.3% power to detect 1° difference of the mechanical axis with SD of 1.5° [10], with type I error of 5%. The inter- and intra-observer reliability based on the intraclass correlation coefficients (ICC) of the MA measurement were 0.842 and 0.918, respectively. The inter- and intra-rater of ICC for tibial and femoral component alignment ranged from 0.746 to 0.818. Statistical analysis was performed with the use of the Stata/MP 15.0 software (StataCorp LP, College Station, TX, USA). The level of significance was set at *P* < 0.05.

## Results

**Fig. 1:**
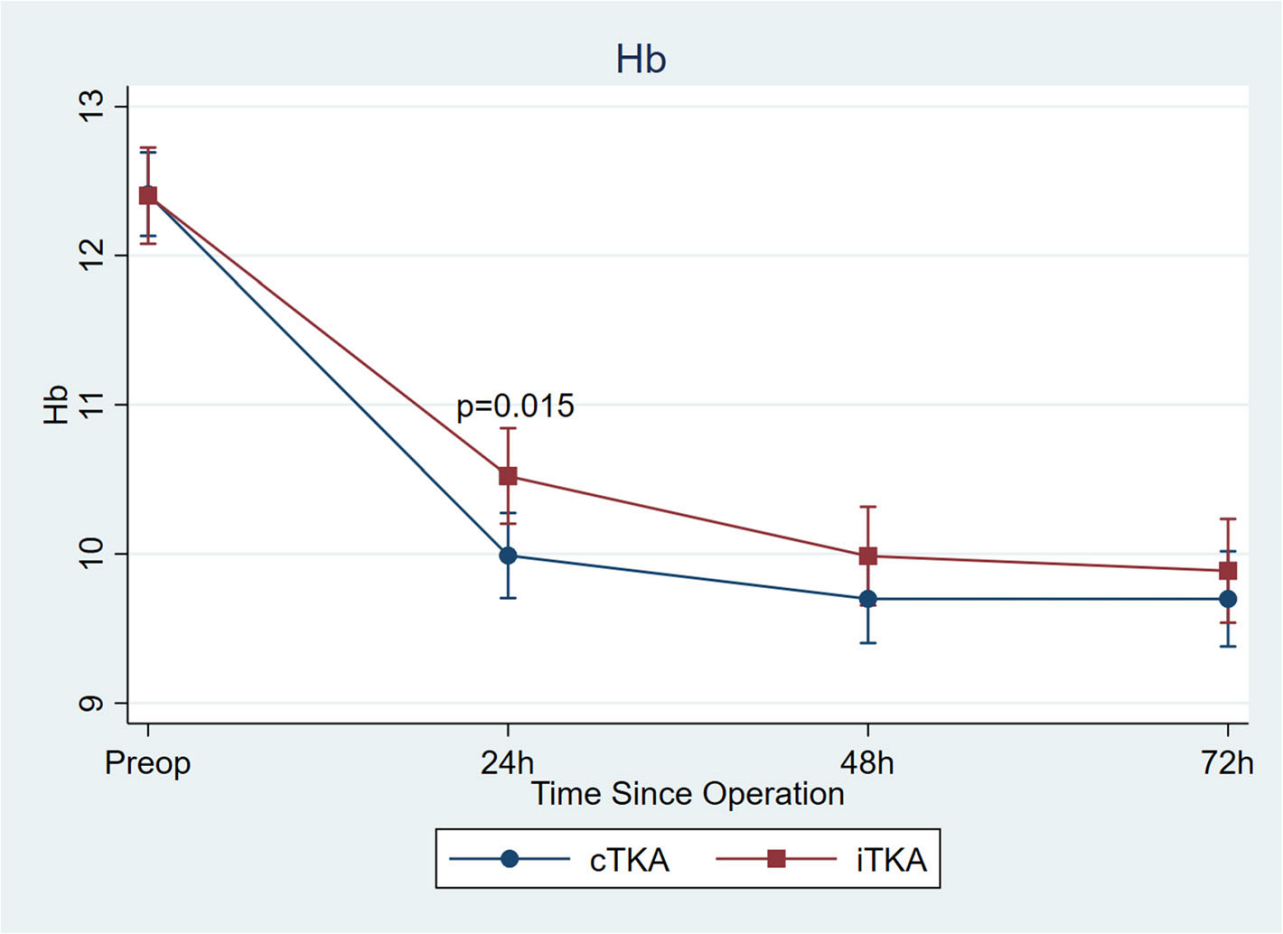
Serum hemoglobin level obtained at preoperative, 24 h, 48 h and 72 h postoperative. Repeated measures ANOVA was used to compare between groups. Data are presented with mean ± 95% confidence interval. * = statistically significant (*p* = 0.02)

Comparing consecutive 44 iBTKA to 57 cBTKA procedures, no differences in the demographic data including age, gender, American Society of Anesthesiologists (ASA) physical status classification, Body mass index (BMI), and preoperative MA, were evident (Table 1). Preoperative Hb was comparable between the cBTKA and iBTKA groups. Postoperative Hb of the cBTKA group was significantly lower than those in the iBTKA group at 24 h (*p* = 0.02), but this was not significantly different thereafter (Fig. 1). Operative duration, total drain volume, CBL, and blood transfusion rate were also not significantly different between the two groups (Table 2).

**Table 1:** Demographic data and preoperative radiograph of the cBTKA and iBTKA groups

	cBTKA group (***N*** = 57)	iBTKA group (***N*** = 44)	***p*** value
**No. of patients**	57	44	
**Age (year)**	62.81 ± 7.91	64.89 ± 6.20	0.15
**Gender (male/female)**	5/52	5/39	0.67
**ASA (I/II/III)**	5/37/15	0/31/13	0.14
**BMI (kg/m**^**2**^**)**	26.81 ± 3.84	26.79 ± 4.28	0.98
**Preoperative mechanical axis (degree)**	5.02 ± 1.09	5.08 ± 0.92	0.66
**Anatomical distal femoral angle (degree)**	97.43 ± 2.01	97.33 ± 2.89	0.74
**Anatomical proximal tibial angle (degree)**	83.49 ± 2.55	84.10 ± 3.25	0.69

Data are presented with mean ± standard deviation (SD.) except for No. of patients, gender

For anatomical distal femoral and proximal tibial angle, the angle >90^o^ was defined as a valgus position, whereas the angle <90^o^ represented a varus position

*ASA* American Society of Anesthesiologists (ASA) physical status classification, *BMI* body mass index, *kg/m*^*2*^ kilogram/meter^2^

**Table 2:** The perioperative and postoperative characteristics of the cBTKA and iBTKA groups

	cBTKA group (***N*** = 57)	iBTKA group (***N*** = 44)	***p*** value
**Operative duration (min)**	127.25 ± 18.95	125.18 ± 12.98	0.54
**Total drain volume** **(ml)**	420.88 ± 240.54	354.55 ± 176.43	0.13
**CBL (ml)**	1052.41 ± 343.87	998.04 ± 311.46	0.42
**Blood transfusion**	43.86%	43.18%	0.95

Data are presented with mean ± SD except blood transfusion rate

*min* minutes, *ml* milliliters, *mg* milligrams, *CBL* calculated blood loss

**Fig. 2:**
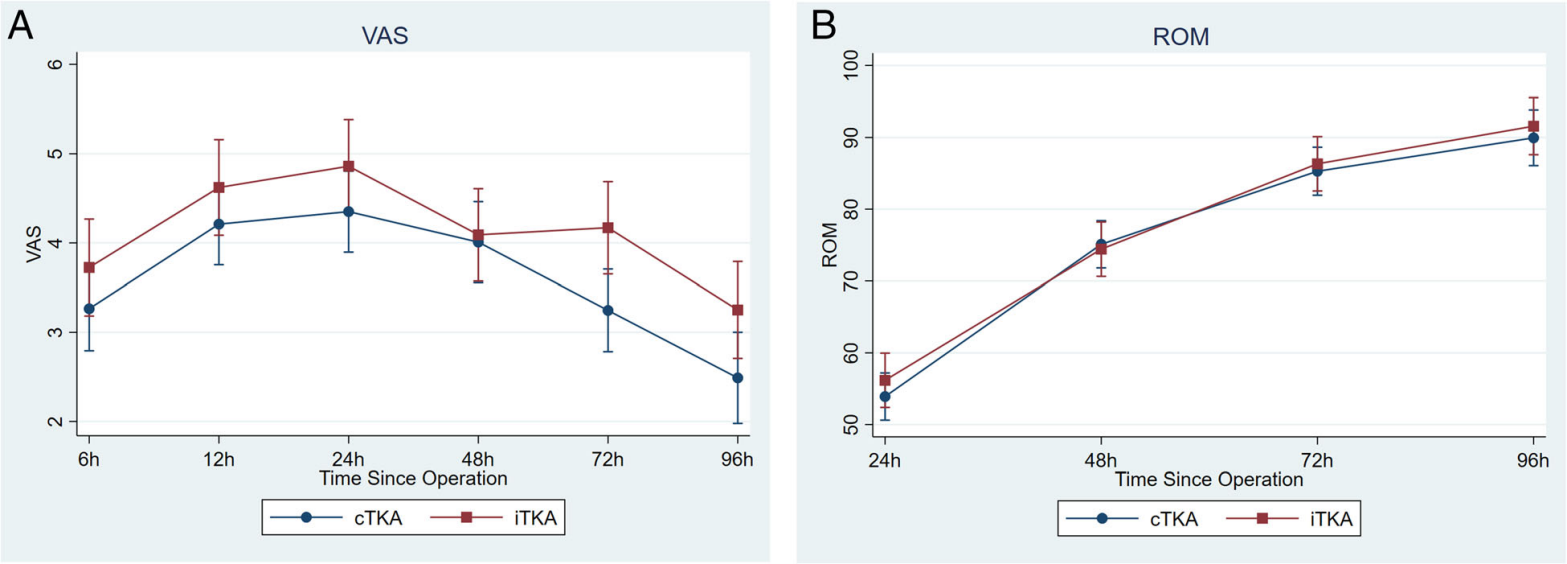
**a** Visual analog scales (VAS) for pain intensity assessed at 6 h, 12 h, 24 h, 48 h and 72 h postoperative. **b** Knee flexion angle assessed at 24 h, 48 h and 72 h postoperative. Repeated measures ANOVA was used to compare between groups. Data are presented with mean ± 95% confidence interval

There was no significant difference in postoperative VAS (Fig. 2a) and knee flexion angle between both groups (Fig. 2b). The cBTKA consumed total morphine of 14.61 ± 9.96 and the iBTKA group 14.30 ± 9.84 mg (*p* = 0.90) at 24 h postoperative, and of 23.01 ± 16.79 and 19.77 ± 13.84 mg respectively (*p* = 0.41) at 48 h. The LOS of iBTKA was 5.43 ± 1.54 days, whereas it was 4.98 ± 1.10 days for cBTKA group (*p* = 0.30).

For radiographic measures, the mean of MA and component orientation in the iBTKA group were more accurate than achieved in the cBTKA group (*p* < 0.01). The iBTKA group also had a significantly lower number of outliers that deviated > ±3° of neutral MA and aiming target of component positioning (except for SFA) (Table 3).

**Table 3:** The postoperative radiographic outcomes of the cTKA and iTKA groups

Variables	cTKA group (***N*** = 57)	iTKa group (***N*** = 44)	***p*** value
**Mechanical axis**	2.29^o^ ± 0.81	1.79^o^ ± 0.85	< 0.01*
**MA outlier**	21.93%	7.95%	0.01*
**CFA**	91.74^o^ ± 1.99	90.60^o^ ± 1.98	< 0.01*
**CFA outlier**	27.19%	11.36%	0.01*
**CTA**	87.77^o^ ± 1.79	89.18^o^ ± 1.85	< 0.01*
**CTA outlier**	25.44%	10.23%	0.01*
**SFA**	89.28^o^ ± 2.68	90.54^o^ ± 2.44	< 0.01*
**SFA outlier**	21.05%	20.45%	1.00*
**STA**	83.40^o^ ± 3.32	85.39^o^ ± 2.60	< 0.01*
**STA outlier**	44.74%	21.59%	< 0.01*

Data are presented with mean ± SD except all outliers

*MA* mechanical axis, *CFA* coronal femoral component angle, *CTA* coronal tibial component angle, *SFA* sagittal femoral component angle, *STA* sagittal tibial component angle or tibial slope

* = statistically significant (*p* < 0.05)

There were no intraoperative surgical complications identified in either group. For the cBKTA group, 1 patient experienced periprosthetic joint infection in unilateral knee, which was successfully treated with 2 staged revision TKA, and 1 had recurrent hemarthrosis in unilateral knee which was successfully treated with 1 staged revision TKA. One patient in the iBTKA group suddenly died at 4 months after full recovery from the surgery because of acute myocardial infarction.

## Discussion

The clinical success of TKA basically depends on patient selection and surgical techniques used. Correct postoperative mechanical and component alignments are the paramount goal to guarantee optimal function and longevity of the implant [22]. In addition, a blood preserving strategy and multimodal pain control could also be additional keys for satisfactory TKA, and may be particularly important for BTKA because of its substantial bone and soft tissue trauma. Our study found comparable blood loss, and blood transfusion, between the iBTKA and cBTKA. As well, no differences between the ABN and conventional TKA regarding early postoperative VAS, morphine consumption, knee flexion angle, LOS, and complication rate, were found.

Prior studies have revealed that ABN can reduce estimate blood loss and Hb dropping after surgery compared to conventional TKA [11, 12]. Ikawa et al. [10] conducted a prospective RCT study and reported that 121 unilateral ABN assisted TKA had 287 ml less average blood loss than 120 unilateral conventional TKA (*p* < 0.05). However, comparable blood loss was proposed by other investigations [23, 24]. A recent systematic review and meta-analysis [8] also revealed an insignificant difference in postoperative blood loss-related indicators between TKA using the ABN and the conventional system. Our results accorded with this as we found no difference between iBTKA and cBTKA in terms of CBL and blood transfusion rate, even if the postoperative Hb of cBTKA group dropped to a significantly lower level than the iBTKA group at 24 h. While preclusion of intramedullary canal violation in iBTKA group might reduce drain volume by approximately 50 ml, it seemed unable to reduce hidden blood loss even in a BTKA setting. Therefore, our finding suggested that using the conventional intramedullary guide might have no significant effect on total blood loss, when both the bone plug that occluded into the femoral opening hole, and the application of intra-articular tranexamic acid (TXA) used in our study may have been effective in mitigating blood loss after BTKA [20]. Additionally, some studies have proposed that bleeding from trimmed cancellous bone of an uncovered resection surface [5, 25, 26] and a soft tissue surface [3] may be more significant sources of bleeding after TKA.

It has also been suggested that intramedullary reaming and insertion of conventional instruments may inevitably lead to the destruction of endothelial cells, and dissipation of marrow emboli, which may subsequently induce a more inflammatory response and risk of embolism-associated morbidities such as acute cardiac disorders [7, 27, 28]. Accordingly, some inflammatory markers have been shown to be associated with functional recovery and morbidities following some major orthopedic surgeries [29–31]. However, our study demonstrated non-superiority of ABN over conventional TKA in regard to early postoperative pain, knee flexion, LOS, and complications.

For lower limb alignment, meta-analysis demonstrated that ABN system significantly improved the accuracy of postoperative MA, and resulted in fewer number of outliers compared to the conventional system [8]. Additionally, the alignment of ABN assisted TKA was not inferior to computer assisted TKA (CAS), and it did not increase risks associated with extra pin sites [9]. In our study, we found, if the ABN system was utilized, significantly better accuracy of MA and component positioning, and better reduction of outlier exceeding ±3° from the neutral mechanical alignment and aiming target of component orientation. Despite statistical significance, the mechanical alignment of the lower extremity that was improved by 0.5° might still be questionable as to its clinical significance, particularly in regard to the longevity of the prostheses. Slevin et al. [32] assessed the whole leg coronal alignment and component position by using 3D-reconstructed computerized tomography scan (CT), and found that those parameters did not have significant correlation with patient outcomes after CAS TKA. Also, the benefit of CAS in terms of TKA survivorship could not be proven even with long-term follow-up of between 14 and 16 years [33]. Similarly, ABN assisted TKA could not yield superiority in clinical outcomes over convention jig-assisted TKA as shown in recent systematic reviews and meta-analyses [8, 34]. This may be due to the fact that ABN does not significantly alter the surgical process used in conventional TKA, but it just provides accurate alignment information during the procedure. Recently, a systematic review by Budhiparama et al. [35] also found conflicting evidence as to whether ABN device decreases the number of outliers or improves the postoperative alignment to a clinically important level. On the other hand, using ABN might be associated with prolonged operative time [8] and additional cost of US$850–$1000 [34]. Research on cost-effectiveness of ABN systems may be in the early stages, and the result is still inconclusive.

It is acknowledged that our study has some limitations. First, this study is retrospective with some inherent limitations related to the study design. However, baseline characteristics were similar in of the iBTKA and cBTKA groups that were consecutively performed by a single surgeon with identical perioperative care. Second, the patients who enrolled in our study were predominantly female, which might be associated with poorer postoperative pain and less blood loss [36, 37]. Nevertheless, recent RCTs and systemic review could not identify gender as a significant predictor affecting blood loss, postoperative pain, and function recovery after TKA [26, 38, 39]. Third, use of PAI, non-steroidal anti-inflammatory drugs (NSAIDs), and topical TXA in our study protocol might be sufficient to reduce postoperative inflammation, pain, opioids consumption, and blood loss [20, 40], whereas different protocols might yield different findings. Lastly, 31.7% (32/101) of our patients had preoperative anemia which was defined as preoperative Hb < 12 g/dl for female and < 13 g/dl for male, and the blood transfusion threshold for BTKA at our institution is 9 g/dl. These might be an explanation for higher blood transfusion rate in the present study compared to previous reports.

## Conclusion

ABN use in BTKA could not reduce blood loss, postoperative pain and complications, and did not improve functional recovery when compared to conventional BTKA. Although the ABN system significantly improved the accuracy of MA and prostheses positioning, and also reduced the number of outliers compared to conventional instruments, it is still controversial as to whether these improvements to lower limb alignment could benefit patients in regard to long-term functional outcomes and implant survival rate.

## Data Availability

The datasets used and/or analyzed during the current study are available from the corresponding author on reasonable request.

## Abbreviations

TKA: Total knee arthroplasty
OA: Osteoarthritis
IM: Intramedullary
EM: Extramedullary
CAS: Computer-assisted surgery
BTKA: Bilateral one-stage TKA
UTKA: Unilateral TKA
CBL: Calculated blood loss
TXA: Tranexamic acid
ABN: Accelerometer-based navigation
MA: Mechanical axis
cBTKA: Patients who had undergone BTKA with conventional instruments
iBTKA: Patients who had undergone BTKA with ABN
PAI: Periarticular injection
PCA: Patient-controlled analgesia
CPM: Continuous passive motion
AP: Anteroposterior
CFA: Coronal femoral component angle
CTA: Coronal tibial component angle
SFA: Sagittal femoral component angle
STA: Sagittal tibial component angle
Hb: Hemoglobin
VAS: Visual analog scale
LOS: Length of stay
TBV: Total blood volume
ICC: Interclass correlation coefficients
ASA: American Society of Anesthesiologists
BMI: Body mass index
CT: Computerized tomography
